# Multicriteria Group Decision Making by Using Trapezoidal Valued Hesitant Fuzzy Sets

**DOI:** 10.1155/2014/304834

**Published:** 2014-07-14

**Authors:** Tabasam Rashid, Syed Muhammad Husnine

**Affiliations:** Department of Sciences and Humanities, National University of Computer and Emerging Sciences, Lahore Campus, Block-B, Faisal Town, Lahore, Pakistan

## Abstract

The concept of trapezoidal valued hesitant fuzzy set is introduced. Notion for distance between any two trapezoidal valued hesitant fuzzy elements is given. Using this proposed distance measure, we extend the technique for order preference by similarity to ideal solution for trapezoidal valued hesitant fuzzy sets. An example is constructed to show usefulness of this extension for multicriteria group decision making, where the opinions about the criteria values are expressed as trapezoidal valued hesitant fuzzy set.

## 1. Introduction

Multicriteria decision making provides an effective framework for comparison based on the evaluation of multiple conflicting criteria. The decision process of selecting a suitable alternative usually considers many factors, for instance, organizational goals, risks, needs, limited resources, benefits, and so forth. Many techniques are available in the literature to handle multicriteria decision making problems. The weighted sum scalarization approach was used in conjunction with three metaheuristic algorithms, differential evolution, chaotic differential evolution, and gravitational search algorithm [1]. These methods are then used to generate the approximate Pareto frontier to the green sand mould system problem. A multiobjective optimization approach was developed to solve nonlinear fuzzy optimization problems and solutions in the Pareto front that correspond with the fuzzy solution of the former fuzzy problem which was expressed in terms of the group of three parameters, optimal solution, degree of satisfaction, and vagueness factor [2]. The hypervolume-driven analytical programming approaches were developed in [3]. This method was applied to the multiobjective design optimization of a real-world photovoltaic based solar powered irrigation system and this problem was multivariate, nonlinear, and multiobjective. Recently a classical approach technique for order preference by similarity to ideal solution (TOPSIS) is developed for multiattribute/multicriteria decision making (MADM/MCDM) problems [4]. TOPSIS is one of the major techniques in dealing with MCDM problems. It simultaneously considers both the smallest distance from the positive-ideal solution (PIS) and the largest distance from the negative-ideal solution (NIS). The ranking of an alternative depends on the ratio of its two-distance measures. The technique is useful for decision makers (DM) to structure the problems, conduct analysis, and rank the alternatives [5]. According to Kim et al. [6], four TOPSIS advantages are addressed: (i) a sound logic that represents the rationale of human choice, (ii) a scalar value that accounts for both the best and the worst alternatives simultaneously, (iii) a simple computation process that can be easily programmed into a spreadsheet, and (iv) the performance measures of all alternatives on attributes that can be visualized on a polyhedron, at least for any two dimensions. Human judgment and preference are often ambiguous and cannot be estimated with exact numeric value. Thus a set of crisp values is not suitable to model real-world situations. Fuzzy set theory is used to handle any imprecision in decision making problems and the ambiguities in information [7].

Recently, a lot of work on fuzzy TOPSIS has been done by many authors [8–18]. The aggregation processes assume that the criteria (attribute) or preferences of DMs are independent. DMs are invited from similar fields for a decision problem so they have similar knowledge, preference, and social status. Aggregation of DMs' opinions is very important in group decision making problems to perform evaluation process in obtaining a single collective decision [19]. Torra [20] extended the concept of fuzzy sets to hesitant fuzzy sets. The notion of hesitant fuzzy sets is extended to the concepts of interval-valued hesitant fuzzy sets and triangular fuzzy hesitant fuzzy sets. Group decision making problems are solved for hesitant fuzzy sets and with aggregation operators in [21–24]. Interval-valued hesitant fuzzy sets have been used in the applications of group decision making in [25–28]. Yu [29] gave the concept of triangular hesitant fuzzy sets and use it for the solution of decision making problems. Fuzzy data is a data type with imprecision or with a source of uncertainty. It is generally more convenient and useful in describing fuzzy data to use fuzzy numbers [30]. Zimmermann [31, Subsubsection 5.3.2] defined the trapezoidal fuzzy numbers. The aim of this paper is to propose trapezoidal valued hesitant fuzzy sets and extend fuzzy TOPSIS for trapezoidal valued hesitant fuzzy sets under the opinion of decision makers. The proposed method is a generalization of all the previous techniques for group decision making in the extended domains of hesitant fuzzy sets. In fact in the proposed method TOPSIS and trapezoidal valued hesitant fuzzy sets are for the first time used simultaneously.

This paper is organized as follows. In Section 2, we review fuzzy sets and concepts of hesitant fuzzy sets (HFS), interval-valued hesitant fuzzy sets (IVHFS), and triangular hesitant fuzzy sets, as well as distance notion for trapezoidal fuzzy numbers. In Section 3, concept of hesitant fuzzy sets is extended to the trapezoidal valued hesitant fuzzy sets (TVHFS). Moreover, we gave a notion of distance measure between any two trapezoidal valued hesitant fuzzy elements. In Section 4, fuzzy TOPSIS is established for TVHFS and flow chart is given for this TOPSIS. Then, in Section 5, modified fuzzy TOPSIS method is applied for the ranking of alternatives in an example to see the feasibility of the proposed fuzzy TOPSIS. Conclusion is given in the last section.

## 2. Preliminaries

Some preliminary concepts are given in this section to explain our proposal.

Let *X* be a crisp universe of generic elements, a* fuzzy set B* in the universe; *X* is a mapping from *X* to [0,1]. For any *x*∈*X*, the value *B*(*x*) is called the* degree of membership of x* in *B*. Torra [20] introduced an extension for fuzzy sets to manage those situations in which several values are possible for the definition of a membership function of a fuzzy set and defined hesitant fuzzy set (HFS) in terms of a function that returns a set of membership values for each element in the domain.

**Definition 1 (see [20]).**

A hesitant fuzzy set (HFS) on *X* is a function *h* that when applied to *X* returns a subset of [0,1]. To be easily understood, Xia and Xu [32] expressed the HFS by a mathematical symbol:

(1) $$\begin{array}{c} E=\left\{\left(x,h\left(x\right)\right)\;|\;x\in X\right\}, \end{array}$$

where *h*(*x*) is a set of some values in [0,1], denoting the possible membership degrees of the element *x* ∈ *X* to the set *E*. For convenience, Xia and Xu [32] named *h*(*x*) a hesitant fuzzy element.

**Definition 2 (see [33]).**

An interval-valued hesitant fuzzy set on *X* is a function *h* that when applied to *X* returns a subset of *D*[0,1], where *D*[0,1] is the set of all closed subintervals of [0,1], which can be represented as the following mathematical symbol:

(2) $$\begin{array}{c} E=\left\{\left(x,h\left(x\right)\right)\;|\;x\in X\right\}, \end{array}$$

where *h*(*x*) is a set of some values in *D*[0,1], denoting the possible membership degrees of the element *x* ∈ *X* to the set *E*. For convenience, *h*(*x*) is named as an interval-valued hesitant fuzzy element.

**Definition 3 (see [29]).**

A triangular hesitant fuzzy set on *X* is a function *h* that when applied to *X* returns a subset of Tri[0,1], where Tri[0,1] is the set of all triangular fuzzy numbers in [0,1], which can be represented as the following mathematical symbol:

(3) $$\begin{array}{c} E=\left\{\left(x,h\left(x\right)\right)\;|\;x\in X\right\}, \end{array}$$

where *h*(*x*) is a set of some values in Tri[0,1], denoting the possible membership degrees of the element *x* ∈ *X* to the set *E*. For convenience, *h*(*x*) is named as a triangular hesitant fuzzy element.

**Definition 4 .**

A function “*A*,” given by

(4) $$\begin{array}{c} A\left(x\right)=\{\begin{array}{cc} 0 & \text{if}\;\;x<a\;\;\text{or}\;\;x>d,\\ \frac{x-a}{b-a} & \text{if}\;\;a\leq x\leq b,\\ 1 & \text{if}\;\;b\leq x\leq c,\\ \frac{x-d}{c-d} & \text{if}\;\;c\leq x\leq d, \end{array} \end{array}$$

where 0 ≤ *a* ≤ *b* ≤ *c* ≤ *d* ≤ 1, is called trapezoidal fuzzy number. Symbolically, *A* is denoted by (*a*, *b*, *c*, *d*) (see [34, p. 330]).

There are several distance and dissimilarity measures between any two trapezoidal fuzzy numbers in literature, like Hathaway et al. [35] distance “*d*_*h*_,” Yang et al. [36, 37] distances “*d*_LR_” and “*d*_*f*_,” Hung et al. [38] distance “*D*_MLR_,” Beg and Rashid [39] distance “d*” and square distance “d**,” and so forth. In this paper we will use the distance “d*” and it is also used in [40].

**Definition 5 (see [39]).**

Hamming distance d* between any two trapezoidal fuzzy numbers is given by

(5) $$\begin{array}{c} d^{*}\left(A,B\right)=\frac{1}{4}\left(\left|a_{1}-a_{2}\right|+\left|b_{1}-b_{2}\right|+\left|c_{1}-c_{2}\right|+\left|d_{1}-d_{2}\right|\right), \end{array}$$

where *A* = (*a*_1_, *b*_1_, *c*_1_, *d*_1_) and *B* = (*a*_2_, *b*_2_, *c*_2_, *d*_2_) are two trapezoidal fuzzy numbers.

## 3. Trapezoidal Valued Hesitant Fuzzy Sets

In this section, we propose the concept of trapezoidal valued hesitant fuzzy set (TVHFS). Elements of TVHFS are known as trapezoidal valued hesitant fuzzy element (TVHFE). TVHFS is a generalization of the concept of triangular hesitant fuzzy set, interval-valued hesitant fuzzy set, and hesitant fuzzy set.

**Definition 6 .**

A trapezoidal valued hesitant fuzzy set on *X* is a function *h* that when applied to *X* returns a subset of Trap[0,1], where Trap[0,1] is the set of all trapezoidal values in [0,1], which can be represented as the following mathematical symbol:

(6) $$\begin{array}{c} E=\left\{\left(x,h\left(x\right)\right)\;|\;x\in X\right\}, \end{array}$$

where *h*(*x*) is a set of some values in Trap[0,1], denoting the possible membership degrees of the element *x* ∈ *X* to the set *E*. For convenience, *h*(*x*) is named as a trapezoidal valued hesitant fuzzy element (TVHFE):

(7) E={(x,(x1,x2,…,xn)) ∣ there  exists  n∈N  for  x∈X}  ={ (x,((a1x,b1x,c1x,d1x),(a2x,b2x,c2x,d2x),…, (anx,bnx,cnx,dnx))) ∣ there  exists  n∈N  for  x∈X}.

A typical TVHFS is a fuzzy set, where *h*(*x*) is a finite subset of Trap[0,1]. Examples of TVHFS are given below, where *h*(*x*) represents the possible membership values of *x* in the set.

It is noted that the number of trapezoidal fuzzy numbers in different TVHFE may be different; let *Ln*_*h*(*x*)_ be the number of trapezoidal fuzzy numbers in *h*(*x*). In case values in a TVHFE are out of order, we can arrange trapezoidal fuzzy numbers in *h*(*x*) such an order, that a TVHFE *h*; let *σ* : (1,2,…, *n*)→(1,2,…, *n*) be a permutation satisfying *a*_*σ*(*i*)_ ≤ *a*_*σ*(*i*+1)_,  *i* = 1,2,…, *Ln*_*h*_ − 1. Two TVHFEs *h*(*x*) and *h*(*y*) have the same length *l* and *a*_*σ*(*i*)*x*_ = *a*_*σ*(*i*)*y*_,  *b*_*σ*(*i*)*x*_ = *b*_*σ*(*i*)*y*_,  *c*_*σ*(*i*)*x*_ = *c*_*σ*(*i*)*y*_, and *d*_*σ*(*i*)*x*_ = *d*_*σ*(*i*)*y*_ if and only if *h*(*x*) = *h*(*y*), for *i* = 1,2,…, *l*.

**Example 7 .**

Let *X* be a reference set; then the following are some TVHFS.

 Empty set: *h*(*x*) = {(0,0, 0,0)} for all *x* in *X*.
 Full set: *h*(*x*) = {(1,1, 1,1)} for all *x* in *X*.
 Complete ignorance for a *x* ∈ *X* (all is possible): *h*(*x*) = Trap[0,1].
 Nonsense for a *x* ∈ *X*:*h*(*x*) = *∅*.

**Example 8 .**

Let *X* = {*x*_1_, *x*_2_} be a reference set; then we consider a hesitant fuzzy set *A* given by

(8)  A={(x1,(0.2,0.23,0.24,0.27),(0.25,0.3,0.33,0.35), (0.6,0.7,0.8,0.9)),(x2,(0.1,0.12,0.15,0.2),(0.4,0.45,0.5,0.55))}.

Motivated by the Hausdorff distance, we give a distance notion for any two TVHFEs and then use this distance in construction of TOPSIS for TVHFS.

**Definition 9 .**

Let *x* and *y* be the two TVHFEs, such that *h*(*x*) = {*x*_1_, *x*_2_,…, *x*_*n*_} and *h*(*y*) = {*y*_1_, *y*_2_,…, *y*_*m*_}, where *x*_*i*_ and *y*_*i*_ are trapezoidal fuzzy numbers; then distance “*d*” between *x* and *y* is defined as

(9) d(x,y)=max⁡{max⁡xi∈h(x){min⁡yi∈h(y)(d∗(xi,yi))},max⁡yi∈h(y){min⁡xi∈h(x)(d∗(xi,yi))}}.

The distance d* is defined in Definition 5 and (5), which is a distance measure between two trapezoidal fuzzy numbers.

It is easy to see that this distance “*d*” satisfies the following properties.

1. *d*(*x*, *y*) = 0 if and only if *x* = *y*;
2. *d*(*x*, *y*) = *d*(*y*, *x*).

## 4. TOPSIS for TVHFS

Multicriteria group decision making problem includes uncertain and imprecise data and ambiguities in information coming from human judgment and preference. Extension of TOPSIS for multicriteria group decision making is given, where the opinions about the criteria values are expressed in TVHFS. Suppose that, in this group decision making problem, *E* = {*e*_1_, *e*_2_,…, *e*_*K*_} is the set of the decision makers involved in the decision problem, *A* = {*A*_1_, *A*_2_,…, *A*_*m*_} is the set of the alternatives, and *C* = {*C*_1_, *C*_2_,…, *C*_*n*_} is the set of the criteria used for evaluating the alternatives.

*Step 1*. Let ${\tilde{X}}^{l}={\left[H_{S_{ij}}^{l}\right]}_{m\times n}$ be a fuzzy decision matrix for the MCDM problem, where performance of alternative *A*_*i*_ with respect to decision maker *e*_*l*_ and criterion *C*_*j*_ is denoted as *H*_*S*_*ij*__^*l*^, in a group decision environment with *K* decision makers.

*Step 2*. Aggregated matrix *X* is calculated by the opinions of DMs $\left({\tilde{X}}^{1},{\tilde{X}}^{2},\ldots ,{\tilde{X}}^{K}\right)$ ;  *X* = [*x*_*ij*_], where *x*_*ij*_ = {*x*∣*x* ∈ *H*_*S*_*ij*__^*l*^, where *s*_*p*_*ij*__ ≤ *a*_*tx*_ and *d*_*tx*_ ≤ *s*_*q*_*ij*__ or *d*_*tx*_ = *s*_*p*_*ij*__ or *a*_*tx*_ = *s*_*q*_*ij*__ for all *l* and *t* = 1,2,…, *Ln*_*H*_*S*_*ij*__^*l*^_}, where

(10) $$\begin{array}{c} s_{p_{ij}}=\min\left\{\overset{K}{\underset{l=1}{\min}}\left(\overset{Ln_{H_{S_{ij}}^{l}}}{\underset{t=1}{\max}}\left(d_{tx}\right)\right),\overset{K}{\underset{l=1}{\max}}\left(\overset{Ln_{H_{S_{ij}}^{l}}}{\underset{t=1}{\min}}\left(a_{tx}\right)\right)\right\},\\ s_{q_{ij}}=\max\left\{\overset{K}{\underset{l=1}{\min}}\left(\overset{Ln_{H_{S_{ij}}^{l}}}{\underset{t=1}{\max}}\left(d_{tx}\right)\right),\overset{K}{\underset{l=1}{\max}}\left(\overset{Ln_{H_{S_{ij}}^{l}}}{\underset{t=1}{\min}}\left(a_{tx}\right)\right)\right\}. \end{array}$$

Performance of alternative *A*_*i*_ with respect to criterion *C*_*j*_ is denoted as *x*_*ij*_, in an aggregated matrix *X*.

*Step 3*. Let Ω_*b*_ be the collection of benefit criteria (i.e., the larger *C*_*j*_, the greater preference) and let Ω_*c*_ be the collection of cost criteria (i.e., the smaller *C*_*j*_, the greater preference). The TVHFS positive-ideal solution (TVHFS-PIS), denoted as ${\tilde{A}}^{+}=\left(\begin{array}{cccc} {\tilde{V}}_{1}^{+} & {\tilde{V}}_{2}^{+} & \cdots & {\tilde{V}}_{n}^{+} \end{array}\right)$ , and the TVHFS negative-ideal solution (TVHFS-NIS), denoted as ${\tilde{A}}^{-}=\left(\begin{array}{cccc} {\tilde{V}}_{1}^{-} & {\tilde{V}}_{2}^{-} & \cdots & {\tilde{V}}_{n}^{-} \end{array}\right)$ , are defined as follows:

(11) A~+=[x ∣ x∈HSijl∀i,max⁡l=1K(max⁡i(min⁡t=1LnHSijlatx))≤atx,dtx≤ max⁡l=1K(max⁡i(max⁡t=1LnHSijldtx)) ∣ j∈Ωb,x ∣ x∈HSijl∀i,min⁡l=1K(min⁡i(min⁡t=1LnHSijlatx))≤atx,dtx≤ min⁡l=1K(min⁡i(max⁡t=1LnHSijldtx)) ∣ j∈Ωc]i=1,2,…,m, j=1,2,…,n,t=1,2,…,LnHSijl,A~+=(V~1+V~2+⋯V~n+),

(12) A~−=[x ∣ x∈HSijl∀i,max⁡l=1K(max⁡i(min⁡t=1LnHSijlatx))≤atx,dtx≤ max⁡l=1K(max⁡i(max⁡t=1LnHSijldtx)) ∣ j∈Ωc,x ∣ x∈HSijl∀i,min⁡l=1K(min⁡i(min⁡t=1LnHSijlatx))≤atx,dtx≤ min⁡l=1K(min⁡i(max⁡t=1LnHSijldtx)) ∣ j∈Ωb]i=1,2,…,m, j=1,2,…,n,t=1,2,…,LnHSijl,A~−=(V~1−V~2−⋯V~n−).

*Step 4*. Construction of positive-ideal separation matrix (*D* ^+^) is defined as follows:

(13) $$\begin{array}{c} D^{+}=\left[\begin{array}{c} d\left(x_{11},{\tilde{V}}_{1}^{+}\right)+d\left(x_{12},{\tilde{V}}_{2}^{+}\right)+\cdots +d\left(x_{1n},{\tilde{V}}_{n}^{+}\right)\\ d\left(x_{21},{\tilde{V}}_{1}^{+}\right)+d\left(x_{22},{\tilde{V}}_{2}^{+}\right)+\cdots +d\left(x_{2n},{\tilde{V}}_{n}^{+}\right)\\ \;\begin{array}{cccc} \vdots \;\;\; & \;\;\vdots \;\;\; & \vdots \;\; & \;\vdots \end{array}\;\\ d\left(x_{m1},{\tilde{V}}_{1}^{+}\right)+d\left(x_{m2},{\tilde{V}}_{2}^{+}\right)+\cdots +d\left(x_{mn},{\tilde{V}}_{n}^{+}\right) \end{array}\right]. \end{array}$$

Construction of negative-ideal separation matrix (*D* ^−^) is defined as follows:

(14) $$\begin{array}{c} D^{-}=\left[\begin{array}{c} d\left(x_{11},{\tilde{V}}_{1}^{-}\right)+d\left(x_{12},{\tilde{V}}_{2}^{-}\right)+\cdots +d\left(x_{1n},{\tilde{V}}_{n}^{-}\right)\\ d\left(x_{21},{\tilde{V}}_{1}^{-}\right)+d\left(x_{22},{\tilde{V}}_{2}^{-}\right)+\cdots +d\left(x_{2n},{\tilde{V}}_{n}^{-}\right)\\ \begin{array}{cccc} \vdots \;\;\; & \;\;\vdots \;\;\; & \vdots \;\; & \;\vdots \end{array}\\ d\left(x_{m1},{\tilde{V}}_{1}^{-}\right)+d\left(x_{m2},{\tilde{V}}_{2}^{-}\right)+\cdots +d\left(x_{mn},{\tilde{V}}_{n}^{-}\right) \end{array}\right]. \end{array}$$

*Step 5*. The relative closeness (RC) of each alternative to the ideal solution is calculated as follows:

(15) $$\begin{array}{c} \text{RC}\left(A_{i}\right)=\frac{D_{i}^{-}}{D_{i}^{+}+D_{i}^{-}},\;i=1,2,\ldots ,m, \end{array}$$

where $D_{i}^{-}=\sum_{j=1}^{n}d(x_{ij},{\tilde{V}}_{j}^{-})$ , $D_{i}^{+}=\sum_{j=1}^{n}d(x_{ij},{\tilde{V}}_{j}^{+})$ , and *d* is defined in (9).

*Step 6*. Rank all the alternatives *A*_*i*_  (*i* = 1,2,…, *m*) according to the closeness coefficient RC(*A*_*i*_); the greater the value RC(*A*_*i*_), the better the alternative *A*_*i*._

**Figure 1:**
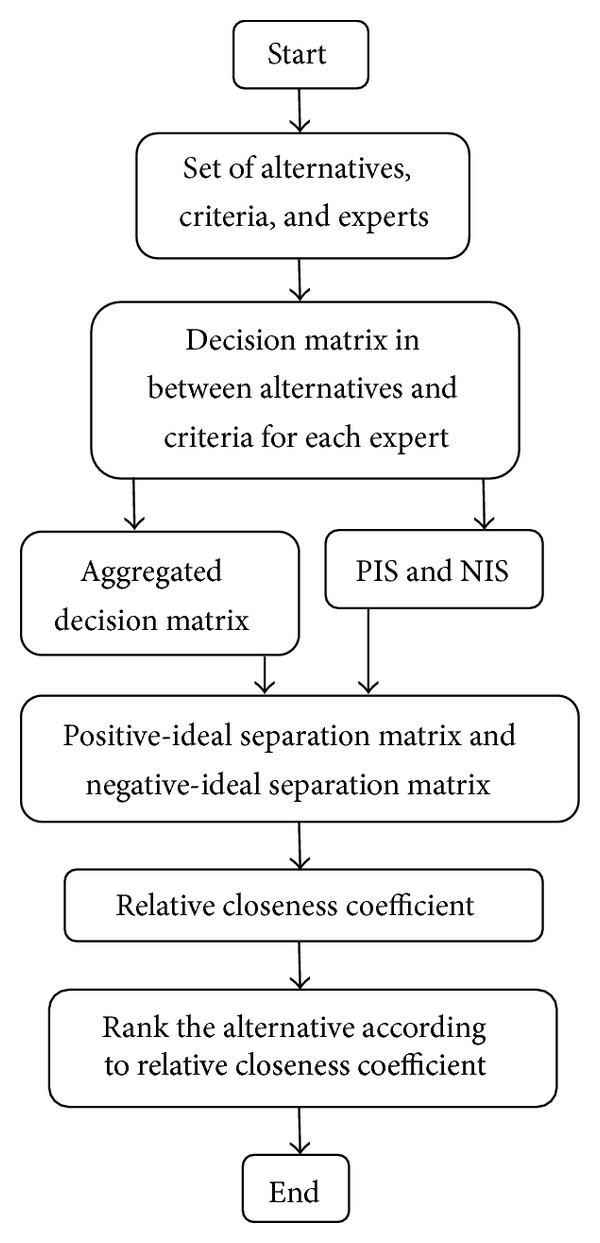
Flow chart of TOPSIS for TVHFS.

A computer-based TOPSIS can be used for this purpose. For the clear illustration of TOPSIS for TVHFS a flow chart is given in Figure 1. The above-mentioned TOPSIS steps are summarized in the following figure.

This proposed procedure helps DMs organize the problems to be solved and carry out analysis, comparisons, and ranking of the alternatives. DMs have the liberty to give different trapezoidal values for the evaluation of criteria for the respective alternatives.

## 5. Illustrative Example

In this section, we give an example. We utilized the method proposed in Section 4 to get the most desirable alternative as well as ranking the alternatives from the best to the worst or vice versa. Five schools (school 1 (*A*_1_), school 2 (*A*_2_), school 3 (*A*_3_), school 4 (*A*_4_), and school 5 (*A*_5_)) all in the field of social sciences are under evaluation by the National Mathematical Foundation (NMF). NMF is a not-for-profit organization which wants to manage the allocation of funds to these schools based on their performance. There are four components (expenses on management of school (*C*_1_), publications from school (*C*_2_), seminar and conference activities by school (*C*_3_), and labs instruments maintenance expense (*C*_4_)) for assessing the capability of five schools.

*Step 1*. There are five possible alternatives *A*_*i*_  (*i* = 1,2, 3,4, 5) to be evaluated on the criteria *C*_*j*_  (*j* = 1,2, 3,4) using the TVHFS by eight decision makers *e*_*K*_  (*K* = 1,2,…, 8), as listed in Tables 1, 2, and 3.

*Step 2*. In Table 4, the decision matrix (*X*) is constructed from Tables 1–3 and by using (10).

*Step 3*. For cost criteria *C*_1_,  *C*_4_ and benefit criteria *C*_2_,  *C*_3_, TVHFS-PIS *A* ^+^ is calculated by utilizing (11) as shown in Table 5.

Similarly, for cost criteria *C*_1_,  *C*_4_ and benefit criteria *C*_2_,  *C*_3_, TVHFS-NIS *A* ^−^ is calculated by utilizing (12) as shown in Table 6.

*Step 4*. By (13), we have positive-ideal matrix (*D* ^+^) which is as follows:

(16) $$\begin{array}{c} D^{+}=\left[\begin{array}{c} 0.33+0.2725+0.6125+0.3025\\ 0.155+0.4075+0.4150+0.4925\\ 0.33+0.3925+0.3975+0.0675\\ 0.6175+0.5025+0.7925+0.605\\ 0.76+0.6025+0.7275+0.8975 \end{array}\right]=\left[\begin{array}{c} 1.5175\\ 1.47\\ 1.1875\\ 2.5175\\ 2.9875 \end{array}\right]. \end{array}$$

Now by (14), we obtain negative-ideal matrix (*D* ^−^) which is as follows:

(17) $$\begin{array}{c} D^{-}=\left[\begin{array}{c} 0.725+0.5125+0.2175+0.725\\ 0.76+0.3575+0.4525+0.4925\\ 0.655+0.3725+0.615+0.8975\\ 0.3475+0.2625+0.0375+0.4925\\ 0.3625+0.2175+0.1025+0.3025 \end{array}\right]=\left[\begin{array}{c} 2.18\\ 2.0625\\ 2.54\\ 1.14\\ 0.985 \end{array}\right]. \end{array}$$

*Step 5*. Relative closeness (RC) of each alternative to the ideal solutions is calculated by using (15):

(18) $$\begin{array}{c} \text{RC}\left(A_{1}\right)=\frac{2.18}{\left(2.18+1.5175\right)}=0.5896;\\ \text{RC}\left(A_{2}\right)=\frac{2.0625}{\left(2.0625+1.47\right)}=0.5839;\\ \text{RC}\left(A_{3}\right)=\frac{2.54}{\left(2.54+1.1875\right)}=0.6814;\\ \text{RC}\left(A_{4}\right)=\frac{1.14}{\left(1.14+2.5175\right)}=0.3117;\\ \text{RC}\left(A_{5}\right)=\frac{0.985}{\left(0.985+2.9875\right)}=0.248. \end{array}$$

*Step 6*. Rank all the alternatives *A*_*i*_  (*i* = 1,2,…, 5) according to the closeness coefficient RC(*A*_*i*_):

(19) $$\begin{array}{c} A_{3}≻A_{1}≻A_{2}≻A_{4}≻A_{5}. \end{array}$$

Thus the most desirable alternative is *A*_3_.

## 6. Discussion

Fuzzy TOPSIS methodologies are a hybrid application of soft computing techniques. The aim of a fuzzy TOPSIS model is to assess the overall cost and benefits of schools in which the descriptions of criteria and their observations are imprecise, vague, and uncertain. This study comprises the selection of schools by assessing their needs and outcomes under vague and uncertain environments. Funds allocation is a multicriteria decision making problem. In fact, one further aim of this study is to develop a decision support system for school selection under the uncertain environment.

## 7. Conclusion

Conventional methods are not useful to convey the imprecision in decision makers opinion and it is also difficult in the domain of ordinary fuzzy set theory. DMs gave their opinions about the criteria of alternatives by TVHFS. The TVHFS is the way to deal with uncertainty in any information. Multicriteria analysis provides an effective framework for evaluation of alternatives. Fuzzy TOPSIS method is proposed for TVHFS to solve multicriteria decision making problem with the opinion of some experts. The relative closeness coefficient has ranked the alternatives from the best to the worst by considering the smallest distance from the positive-ideal solution (PIS) and also the largest distance from the negative-ideal solution (NIS). A modified fuzzy TOPSIS produces satisfactory results by providing positive-ideal separation and negative-ideal separation matrices. A numerical example is given for the ranking of alternatives to show the feasibility of our proposed fuzzy TOPSIS for multicriteria group decision making. This proposed method is different from all the previous techniques of group decision making due to the fact that this method uses TOPSIS and TVHFS simultaneously. It is efficient for real-world decision making applications, like robot selection, location selection, and medical diagnostics. In the future we plan to study Choquet integral based TOPSIS for TVHFS. Furthermore, algebraic operations for TVHFS will also be developed.

## Figures and Tables

**Table 1:** Decision matrix ( ${\tilde{X}}^{1}$ ) with respect to decision makers 1, 2, and 3 (*e*_1_, *e*_2_, and  *e*_3_).

*C*_1_	*A*_1_	{(0.45,0.5,0.55,0.6), (0.5,0.55,0.6,0.7), (0.65,0.7,0.75,0.8)}
	*A*_2_	{(0.05,0.12,0.15,0.19), (0.2,0.24,0.29,0.31)}
	*A*_3_	{(0.45,0.5,0.55,0.6), (0.5,0.55,0.6,0.7)}
	*A*_4_	{(0.5,0.55,0.6,0.7), (0.7,0.8,0.85,0.9)}
	*A*_5_	{(0.9,0.95,0.97,1)}

*C*_2_	*A*_1_	{(0.5,0.53,0.64,0.7), (0.65,0.72,0.74,0.8)}
	*A*_2_	{(0.5,0.53,0.64,0.7), (0.65,0.72,0.74,0.8), (0.77,0.8,0.82,0.85)}
	*A*_3_	{(0.5,0.53,0.64,0.7), (0.52,0.55,0.6,0.63)}
	*A*_4_	{(0.05,0.1,0.15,0.2), (0.19,0.21,0.25,0.29)}
	*A*_5_	{(0.05,0.1,0.15,0.2), (0.28,0.31,0.34,0.36)}

*C*_3_	*A*_1_	{(0.05,0.1,0.15,0.2), (0.21,0.24,0.29,0.32)}
	*A*_2_	{(0.42,0.45,0.5,0.55), (0.54,0.57,0.6,0.63)}
	*A*_3_	{(0.64,0.68,0.72,0.75), (0.83,0.88,0.9,0.93)}
	*A*_4_	{(0.05,0.1,0.14,0.19), (0.26,0.3,0.34,0.37)}
	*A*_5_	{(0,0.05,0.1,0.12), (0.14,0.18,0.2,0.23)}

*C*_4_	*A*_1_	{(0.05,0.1,0.15,0.2), (0.21,0.24,0.29,0.32)}
	*A*_2_	{(0.44,0.48,0.5,0.53), (0.55,0.58,0.6,0.62)}
	*A*_3_	{(0.05,0.09,0.1,0.14), (0.16,0.18,0.2,0.23)}
	*A*_4_	{(0.44,0.48,0.5,0.53), (0.55,0.58,0.6,0.62), (0.65,0.68,0.7,0.72)}
	*A*_5_	{(0.32,0.37,0.4,0.43), (0.65,0.68,0.7,0.72)}

**Table 2:** Decision matrix ( ${\tilde{X}}^{2}$ ) with respect to decision makers 4, 5, and 6 (*e*_4_, *e*_5_, and  *e*_6_).

*C*_1_	*A*_1_	{(0.05,0.1,0.15,0.2), (0.19,0.21,0.25,0.27)}
	*A*_2_	{(0,0.02,0.05,0.09), (0.16,0.19,0.2,0.23)}
	*A*_3_	{(0.35,0.39,0.41,0.44), (0.54,0.58,0.6,0.63)}
	*A*_4_	{(0.54,0.58,0.6,0.63), (0.93,0.98,1, 1)}
	*A*_5_	{(0.44,0.48,0.5,0.54), (0.64,0.67,0.7,0.72)}

*C*_2_	*A*_1_	{(0.35,0.39,0.41,0.44), (0.84,0.87,0.9,0.92)}
	*A*_2_	{(0.05,0.08,0.1,0.1), (0.24,0.28,0.3,0.32)}
	*A*_3_	{(0.05,0.1,0.15,0.2), (0.19,0.21,0.25,0.29)}
	*A*_4_	{(0.35,0.39,0.41,0.44), (0.65,0.68,0.7,0.72)}
	*A*_5_	{(0.35,0.39,0.41,0.44), (0.54,0.58,0.6,0.63)}

*C*_3_	*A*_1_	{(0,0.02,0.05,0.09), (0.16,0.19,0.2,0.23)}
	*A*_2_	{(0.35,0.39,0.41,0.44), (0.43,0.47,0.5,0.52)}
	*A*_3_	{(0.35,0.39,0.41,0.44), (0.54,0.58,0.6,0.63)}
	*A*_4_	{(0,0.02,0.05,0.09), (0.05,0.08,0.1,0.1)}
	*A*_5_	{(0,0.02,0.05,0.09), (0.05,0.08,0.1,0.1)}

*C*_4_	*A*_1_	{(0.35,0.39,0.41,0.44), (0.54,0.58,0.6,0.63)}
	*A*_2_	{(0.54,0.58,0.6,0.63), (0.96,1, 1,1)}
	*A*_3_	{(0,0.02,0.05,0.09), (0.16,0.19,0.2,0.23)}
	*A*_4_	{(0.44,0.47,0.5,0.51), (0.68,0.7,0.7,0.72)}
	*A*_5_	{(0.54,0.58,0.6,0.63), (0.98,0.99,0.99,1)}

**Table 3:** Decision matrix ( ${\tilde{X}}^{3}$ ) with respect to decision makers 7 and 8 (*e*_7_, and  *e*_8_).

*C*_1_	*A*_1_	{(0.37,0.4,0.42,0.45), (0.56,0.58,0.6,0.6)}
	*A*_2_	{(0.26,0.29,0.3,0.31), (0.55,0.58,0.6,0.62)}
	*A*_3_	{(0.08,0.1,0.1,0.13), (0.27,0.3,0.3,0.33)}
	*A*_4_	{(0.56,0.6,0.62,0.65), (0.88,0.9,0.92,0.95)}
	*A*_5_	{(0.47,0.5,0.5,0.52), (0.56,0.59,0.6,0.62)}

*C*_2_	*A*_1_	{(0.56,0.6,0.6,0.62), (1,1, 1,1)}
	*A*_2_	{(0.08,0.1,0.1,0.13), (0.27,0.3,0.3,0.33)}
	*A*_3_	{(0.56,0.6,0.62,0.65), (0.88,0.9,0.92,0.95)}
	*A*_4_	{(0.47,0.5,0.5,0.52), (0.68,0.7,0.7,0.74)}
	*A*_5_	{(0.08,0.1,0.1,0.13), (0.27,0.3,0.3,0.33)}

*C*_3_	*A*_1_	{(0.27,0.3,0.3,0.33), (0.47,0.5,0.5,0.52)}
	*A*_2_	{(0.47,0.5,0.5,0.52), (0.88,0.9,0.92,0.95)}
	*A*_3_	{(0.27,0.3,0.3,0.33), (0.68,0.7,0.7,0.74)}
	*A*_4_	{(0,0.02,0.04,0.05), (0.15,0.17,0.2,0.22), (0.35,0.37,0.4,0.41)}
	*A*_5_	{(0.15,0.17,0.2,0.22), (0.35,0.37,0.4,0.41)}

*C*_4_	*A*_1_	{(0,0.02,0.04,0.05), (0.27,0.3,0.3,0.33)}
	*A*_2_	{(0.27,0.3,0.3,0.33), (0.47,0.5,0.5,0.52)}
	*A*_3_	{(0,0.02,0.04,0.05), (0.06,0.1,0.1,0.12)}
	*A*_4_	{(0.47,0.5,0.5,0.52), (0.56,0.57,0.6,0.63), (0.78,0.8,0.8,0.83)}
	*A*_5_	{(0.97,1, 1,1)}

**Table 4:** Decision matrix (*X*).

*C*_1_	*A*_1_	{(0.19,0.21,0.25,0.27), (0.37,0.4,0.42,0.45), (0.45,0.5,0.55,0.6)}
	*A*_2_	{(0.16,0.19,0.2,0.23), (0.26,0.29,0.3,0.31)}
	*A*_3_	{(0.27,0.3,0.3,0.33), (0.35,0.39,0.41,0.44), (0.45,0.5,0.55,0.6)}
	*A*_4_	{(0.56,0.6,0.62,0.65), (0.7,0.8,0.85,0.9)}
	*A*_5_	{(0.56,0.59,0.6,0.62), (0.64,0.67,0.7,0.72), (0.9,0.95,0.97,1)}

*C*_2_	*A*_1_	{(0.56,0.6,0.6,0.62), (0.65,0.72,0.74,0.8)}
	*A*_2_	{(0.24,0.28,0.3,0.32), (0.5,0.53,0.64,0.7)}
	*A*_3_	{(0.19,0.21,0.25,0.29), (0.56,0.6,0.62,0.65)}
	*A*_4_	{(0.19,0.21,0.25,0.29), (0.35,0.39,0.41,0.44), (0.47,0.5,0.5,0.52)}
	*A*_5_	{(0.27,0.3,0.3,0.33), (0.35,0.39,0.41,0.44)}

*C*_3_	*A*_1_	{(0.16,0.19,0.2,0.23), (0.27,0.3,0.3,0.33)}
	*A*_2_	{(0.43,0.47,0.5,0.52), (0.47,0.5,0.5,0.52)}
	*A*_3_	{(0.27,0.3,0.3,0.33), (0.54,0.58,0.6,0.63), (0.64,0.68,0.72,0.75)}
	*A*_4_	{(0,0.02,0.04,0.05), (0.05,0.08,0.1,0.1), (0.05,0.1,0.14,0.19)}
	*A*_5_	{(0.05,0.08,0.1,0.1), (0.15,0.17,0.2,0.22)}

*C*_4_	*A*_1_	{(0.21,0.24,0.29,0.32), (0.35,0.39,0.41,0.44)}
	*A*_2_	{(0.47,0.5,0.5,0.52), (0.54,0.58,0.6,0.63)}
	*A*_3_	{(0.05,0.09,0.1,0.14), (0.06,0.1,0.1,0.12)}
	*A*_4_	{(0.47,0.5,0.5,0.52), (0.55,0.58,0.6,0.62), (0.65,0.68,0.7,0.72), (0.68,0.7,0.7,0.72)}
	*A*_5_	{(0.65,0.68,0.7,0.72), (0.97,1, 1,1)}

**Table 5:** *A* ^+^.

*C*_1_	{(0,0.02,0.05,0.09), (0.05,0.12,0.15,0.19), (0.05,0.1,0.15,0.2), (0.08,0.1,0.1,0.13), (0.16,0.19,0.2,0.23)}

*C*_2_	{(0.56,0.6,0.6,0.62), (0.56,0.6,0.62,0.65), (0.65,0.68,0.7,0.72), (0.65,0.72,0.74,0.8), (0.68,0.7,0.7,0.74), (0.77,0.8,0.82,0.85), (0.88,0.9,0.92,0.95), (1,1, 1,1)}

*C*_3_	{(0.64,0.68,0.72,0.75), (0.83,0.88,0.9,0.93), (0.88,0.9,0.92,0.95)}

*C*_4_	{(0,0.02,0.05,0.09), (0,0.02,0.04,0.05), (0.06,0.1,0.1,0.12)}

**Table 6:** *A* ^−^.

*C*_1_	{(0.9,0.95,0.97,1), (0.93,0.98,1, 1)}

*C*_2_	{(0.05,0.1,0.15,0.2), (0.05,0.08,0.1,0.1), (0.19,0.21,0.25,0.29)}

*C*_3_	{(0,0.02,0.05,0.09), (0,0.02,0.04,0.05), (0.05,0.08,0.1,0.1)}

*C*_4_	{(0.97,1, 1,1), (0.98,0.99,0.99,1)}
